# A randomized controlled efficacy study of the Medido medication dispenser in Parkinson’s disease

**DOI:** 10.1186/s12877-019-1292-y

**Published:** 2019-10-16

**Authors:** K. Hannink, L. ter Brake, N.G.M. Oonk, A.A. Wertenbroek, M. Piek, L. Vree-Egberts, M.J. Faber, J. van der Palen, L.D. Dorresteijn

**Affiliations:** 1 Department of Neurology, Medisch Spectrum Twente, Koningsplein 1, 7512 KZ Enschede, the Netherlands; 2 Department of Neurology, Ziekenhuis Groep Twente, Almelo, the Netherlands; 3 Radboud Institute for Health Sciences, Radboud University Medical Center, Nijmegen, the Netherlands; 4 Department of Epidemiology, Medisch Spectrum Twente, Enschede, the Netherlands; 5 Department of Research Methodology, Measurement, and Data Analysis, University of Twente, Enschede, the Netherlands

**Keywords:** Parkinson’s disease, Electronic medication dispenser, Therapy adherence, Physical disability, Quality of life

## Abstract

**Background:**

Complex medication schedules in Parkinson’s disease (PD) result in lower therapy adherence, which contributes to suboptimal therapy and clinical deterioration. Medication reminder systems might improve therapy adherence and subsequently improve symptoms of PD. This randomized controlled study assessed the effect of the electronic medication dispenser Medido on physical disability in PD, as a proxy for changes in therapy adherence.x

**Methods:**

Eighty-seven patients were randomized into the Medido group or control group. The primary outcome of physical disability was measured by the AMC Linear Disability Scale (ALDS). Secondary outcomes were quality of life (QoL) (PDQ-39), health status (EQ5D-5L, VAS), non-motor symptoms (NMS-Quest), and QoL of the caregiver (PDQ-carer). Measurements were performed at baseline, and after 3 and 6 months follow-up.

**Results:**

When using the Medido, a non-significant improvement of 3.0 points (95% CI -5.6;11.6) was seen in ALDS. The exploratory subgroup Hoehn & Yahr classification (H&Y) > 2.5 improved significantly on ALDS with 14.7 points (95% CI -28.5;-0.9, *p* = 0.029 for group x time interaction). QoL deteriorated with 1.0 point in PDQ-39 (*p* = 0.01 for group x time interaction) in favor of the control group. Non-significant differences were observed for VAS (0.4 points, *p* = 0.057) and NMS-Quest (1.3 points, *p* = 0.095) in favor of the Medido group. No changes over time were observed in EQ5D-5L and PDQ-carer.

**Conclusions:**

Based on these data, no firm conclusion can be drawn, but use of the Medido medication dispenser may result in a clinical improvement of physical disability and seems particularly appropriate for more severe patients.

**Trial registration:**

NTR3917. Registered 19 March 2013.

## Background

Fractionating medication in Parkinson’s Disease (PD) and the use of long-acting dopamine may allow more consistent control of motor symptoms in on-off fluctuations and dyskinesias [1].

Frequent medication modifications and fractionated medication make it hard to adhere to the medication regimen. A substantial proportion (29–67%) of PD patients is not compliant [2–4], and timing non-adherence was the most frequently reported medication error [5].

Non-adherence in turn is a potential risk for unnecessary modifications of medication regimens, because of lower observed efficacy of treatment [6, 7]. Disease duration, polypharmacy, complex medication schedules, misunderstandings, and fear of side effects are reasons for suboptimal therapy adherence [6–9]. However, in PD, age-related factors such as physical difficulties and declining cognition are considered to be even more predictive [10].

Various methods to improve therapy adherence can be considered. According to a comprehensive Cochrane review, most of these methods are complex and not very effective [11]. For PD, the most important method to optimize adherence is improving the ease of administration of the treatment regimen by obtaining a constant stimulation of central dopamine receptors [12]. Innovative solutions increasingly become available to support easy administration and facilitate therapy adherence, though evaluation studies are currently lacking. Because therapy adherence is almost impossible to measure reliably, we used changes in clinical outcomes as a proxy for changes in therapy adherence.

The aim of this randomized controlled trial was to examine the efficacy of the Medido, an electronic medication dispenser, versus regular care, in patients with PD who have four or more medication moments daily and experience on-off fluctuations, regarding physical disability, measured with the AMC Linear Disability Scale (ALDS).

## Methods

### Design

The study was designed as a randomized controlled open label multi-center trial. After providing informed consent, patients and their caregivers were randomly allocated to the intervention group or the control group, using block sizes of four. The intervention group received the Medido, while the control group continued their usual care. Data were obtained at baseline, after 3 months and 6 months follow-up by validated questionnaires.

Ethical approval for this randomized controlled trial was granted by the Medical Ethics Committee Twente, the Netherlands (reference number NL43868.044.13). This trial was performed according to CONSORT guidelines and is registered at www.trialregister.nl: NTR3917**.**

### Randomization

The study used a randomization list, generated using a random number generator, to ensure no prior knowledge of which group the next subject would be randomized to [13]. The randomization list was generated by one of the hospitals’ epidemiologists JvdP. The randomization list was kept separate from the investigators. Consecutive patients from the outpatient clinic were enrolled by LtB, MP and NO. Each new patient was assigned to intervention or control based on the randomization list.

### Participants

Participants were recruited from the department of Neurology in the participating hospital (Medisch Spectrum Twente, Ziekenhuis Groep Twente). Eligibility criteria included: a diagnosis of PD, according to the UK Brain Bank criteria, confirmed by a neurologist; age 40 years or older; a minimum of four moments of medication intake each day, and; experiencing on-off fluctuations. The exclusion criteria were being unable to administer their own medication (i.e. when medication was administered by external home care) and being unable to hear and see the visual and auditory signal of the Medido.

### Intervention

**Fig. 1:**
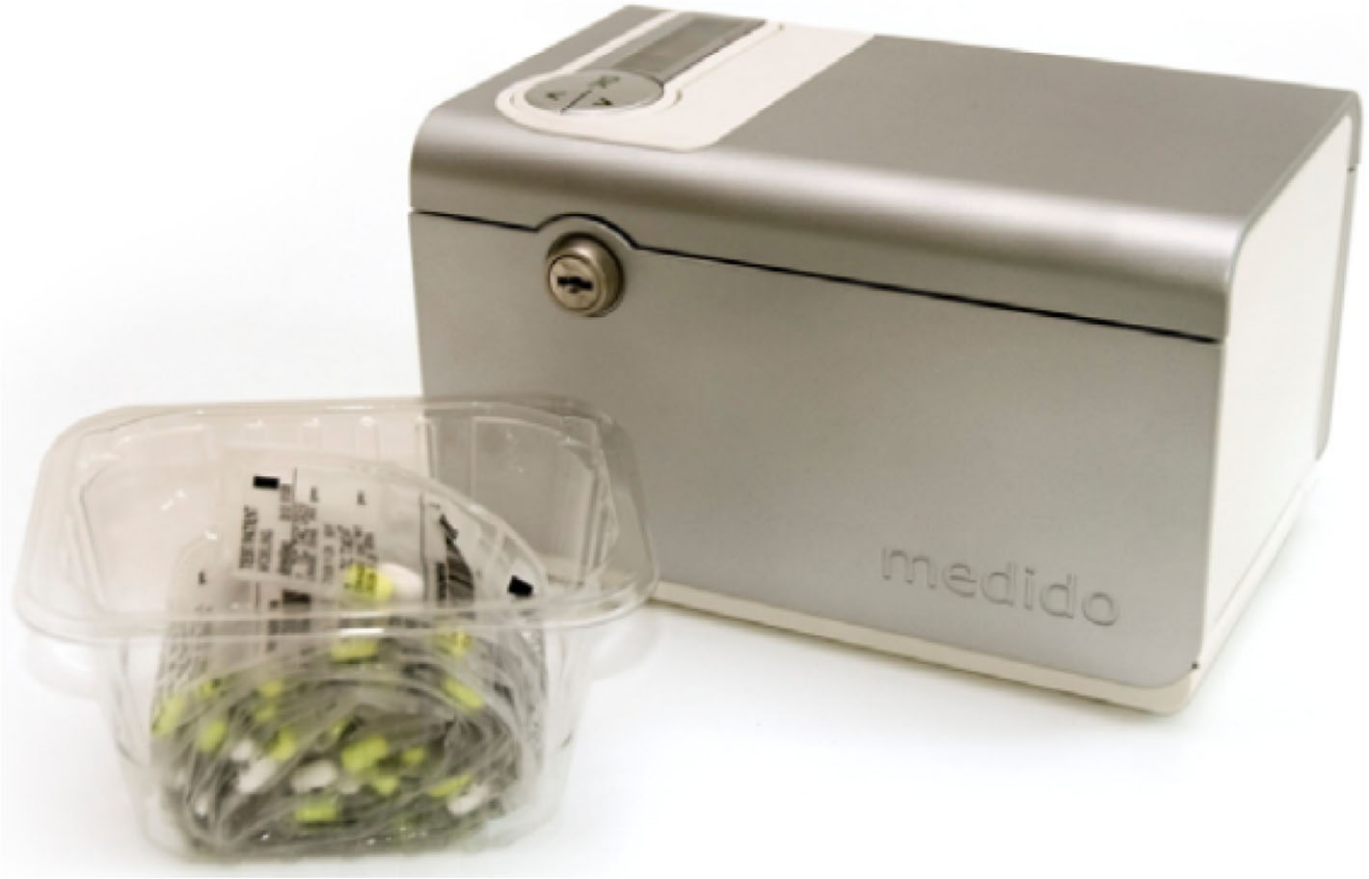
The Medido medication dispenser. Source: Innospense®. Image freely available

The Medido Connected (Innospense BV®, The Hague, the Netherlands) [14] is an electronic medication dispenser (Fig. 1), with a size of 225 mm × 140 mm × 140 mm. It contains pre-packaged medication. At pre-programmed times, the Medido conveys a visual and auditory signal and pre-packaged medication is opened and provided to the user for ingestion of the tablets. If the signal is not acknowledged by the patient within a specified time slot, a message will be sent to the medical caregiver. Therefore, the dispenser is continuously in contact with the internet portal of Innospense®.

### Levodopa equivalent dose (LED)

Possible significant differences between the control group and Medido group in medication regimen at baseline and modifications after 6 months follow-up, were taken into account. Therefore, the LED was used, which calculates the daily dose of levodopa [15].

### Primary outcome

#### Functional disability

The ALDS questionnaire is a generic scale to quantify functional disability. It includes items concerning activities of daily living (ADL), ordered from basic (e.g., self-care, eating) to complex (e.g., household tasks, travelling) performance. The score ranges from 0 to 100, in which a higher score indicates a better functional ability [16]. The 26-item version was used, which is particularly relevant to this population and has adequate clinometric properties for the expected range of disability in patients with PD.

### Secondary outcomes

#### Quality of life

The PDQ-39 questionnaire has 39 items, covering eight discrete domains on QoL in PD. The domains are mobility, ADL, emotional wellbeing, stigma, social support, cognition, communication and physical wellbeing. A higher score means a worse situation [17]. To make valid comparisons to other patient groups and to assess the impact of the disease on QoL, the generic EQ5D-5L questionnaire was used. It comprises five questions on mobility, self-care, pain, usual activities and psychological status. Scores are converted to a score between 1 and zero. A higher score means a better situation. The EQ5D-5L visual analogue score (VAS) was used indicate an overall health related quality of life (HRQoL) score, ranging from 0 (worst imaginable health state) to 100 (best imaginable health state) [18].

#### Non-motor complications

The NMS-Quest is a 30-item questionnaire, covering the domains: gastrointestinal tract, urinary tract, sexual function, cardiovascular, apathy/attention/memory, hallucinations, depression/anxiety, sleep/fatigue and miscellaneous. The more questions answered with ‘present’, the worse the outcome [19].

#### QoL in caregivers

The PDQ-carer questionnaire is a validated 29-item measurement of HRQoL for caregivers of PD patients. The instrument has four domains: social and personal activities, anxiety and depression, self-care, and stress. A higher score means a worse QoL [20].

### Exploratory analyses

Exploratory sub analyses were performed to investigate patient characteristics that could possibly influence questionnaire outcomes. Comparisons were based on the characteristics: Hoehn & Yahr classification (≤ 2.5 versus > 2.5), disease duration (time since diagnosis in years) (< 5 versus ≥5 years), age (< 70 versus ≥70 years of age) and receiving help from a caregiver or not.

### Statistical analysis

Chi-square tests or Fisher’s exact tests, as appropriate, were used to analyze differences between groups in categorical variables and T-tests or Mann-Whitney U tests for continuous variables. Normality of the data was visually inspected.

Score differences between baseline and follow-up were used for analysis of normally distributed data, in order to correct for baseline differences. Thereby considering difference scores would not be influenced, since there was no impact of ceiling or bottom effects in the scores of the different questionnaires. Questionnaires with normally distributed score differences were analyzed by mixed model repeated measurements analysis to take the correlation between repeated measurements on the same patient and random missing data in incomplete repeated questionnaires into account. According to the protocol of the PDQ-39, Expectation Maximization was used to replace random missing data in incomplete questionnaires. Non-normally distributed difference scores were analyzed with a Mann-Whitney-U test.

Cohen’s d effect for the ALDS was calculated as (the difference between two means) / (SD of the ALDS score in the Medido group at baseline). A moderate effect size of 0.5 in the ALDS was considered an important difference [21], given that Cohen classified effect size as small (d = 0.2), medium (d = 0.5) or large (d ≥ 0.8).

Analysis of data was based on the initially assigned treatment after randomization. We used a ‘Modified Intention to Treat’ analysis, including only patients who actually started the study, i.e. those with at least one medication dispensed release from the Medido and a baseline visit at the hospital for the control group.

SPSS version 22 was used for all statistical analysis and *P*-values ≤0.05 were regarded as statistically significant.

### Sample size

In a previous pilot study in a group of patients using the Medido, an increase of 4 points (SD 7 points) on the ALDS questionnaire after 8 weeks follow-up was observed, which was defined as clinically relevant by the developers of the ALDS [16]. When continuing regular care (control group), ALDS scores were expected to remain the same. With α = 5% and a power of 80%, a sample size of 49 patients per group was needed to be able to detect an increase of 4 points with SD = 7. Thus, assuming a drop-out rate of 10%, 110 patients needed to be included in the study.

## Results

### Dataset

**Fig. 2:**
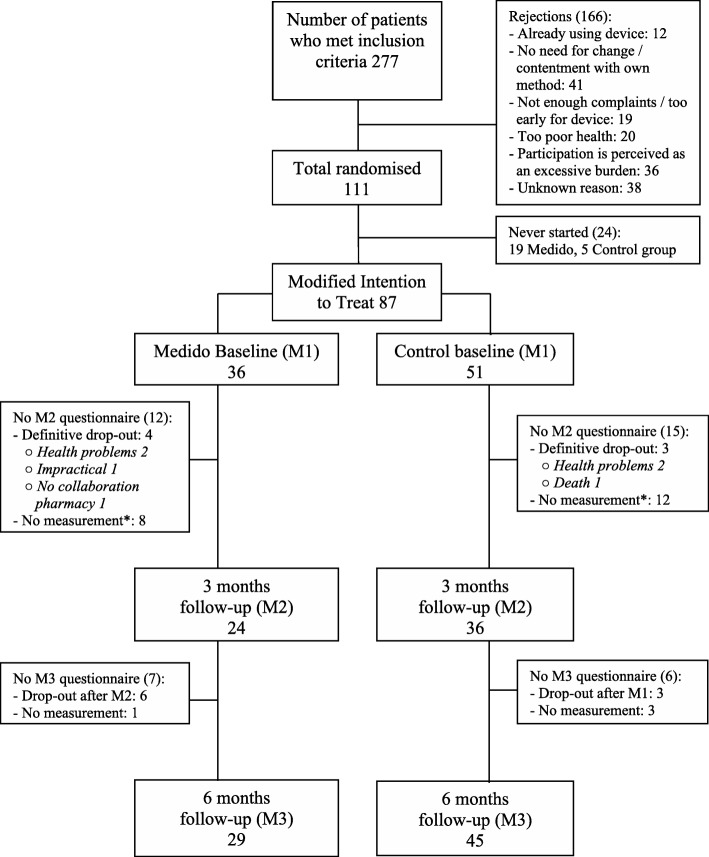
Flow-chart of the study. *due to logistic problems (illness of the senior researcher)

Between May 2013 and July 2014, 277 patients were screened for participation. Figure 2 illustrates the flow chart of the study. Of the 111 randomized patients, only 87 patients and their caregivers ended up in the Modified Intention to Treat analysis, due to the fact that the other patients never actually started using the Medido Connected device (Medido group; *N* = 19) or did not show up for a baseline visit at the hospital (control group; *N* = 5) after randomization.

### Demographic data

Table 1 shows demographic data. There were no significant between-group differences. Age ranged from 42 to 87 years and 39 patients (45%) had a Hoehn & Yahr score >  2.5, indicating more advanced disease.

**Table 1:** Demographic data at baseline

Demographic data		Medido (*n* = 36)	Control (*n* = 51)	Sign. Difference between groups
Gender (n (%))	Male	24 (67)	35 (69)	*p* = 0.847
Age (mean (SD))	Years	69 (7)	68 (11)	*p* = 0.482
Hoehn & Yahr (n (%))	≤ 2.5	18 (50)	30 (59)	*p* = 0.415
	> 2.5	18 (50)	21 (41)	
Disease duration (n (%))	0–5 years	16 (44)	17 (33)	*p* = 0.159
	5–10 years	9 (15)	23 (45)	
	≥ 10 years	11 (31)	11 (22)	
Caregiver available (n (%))	Yes	25 (69%)	33 (65%)	*p* = 0.644
Levodopa Equivalent Dose (mean (SD))	Baseline ΔBL-6 months	851 (415)	799 (331)	*p* = 0.546
		69 (98)	68 (136)	*p* = 0.947

Total dataset of 87 patients, compared by group. Data are presented as numbers (%) or mean (SD).

### Questionnaires

#### Primary outcome

##### ALDS

ALDS scores at baseline were not normally distributed. Therefore, we used normally distributed difference scores between baseline and follow-up. After 6 months of follow-up, the mean score in the Medido group improved by 2.5 points (SE 3.4) (Table 2). Compared to the control group, this resulted in a non-significant difference of 2.9 points (95%CI − 11.6;5.7) in favor of the Medido group. The effect size was small at 0.13.

**Table 2:** Difference scores in ALDS questionnaire data

ALDS [0–100]	Medido			Control			Effect M - C	*P*-value of Difference scores ^a^	Effect Size^b^
	BL (*n* = 36)	ΔBL-3 months (*n* = 24)	ΔBL-6 months (*n* = 29)	BL (*n* = 51)	ΔBL-3 months (*n* = 36)	ΔBL-6 months (*n* = 45)	Effect (95% CI)		
All patients	70.1 (3.9)	1.1 (2.6)	2.5 (3.4)	78.9 (3.3)	2.7 (2.1)	−0.4 (2.7)	2.9 (−11.6;5.7)	0.285	
H&Y ≤ 2.5	83.0 (4.5)	0.8 (2.7)	−3.8 (4.2)	81.4 (3.5)	2.6 (2.2)	2.4 (3.3)	−6.2 (− 4.5;16.9)	0.390	0.43
H&Y > 2.5	57.3 (6.1)	1.5 (4.8)	10.7 (5.3)	75.3 (5.7)	2.7 (4.0)	−4.1 (4.3)	14.7 (−28.5;-0.9)	0.029	0.59
Disease duration < 5	77.1 (5.4)	−5.8 (2.9)	**−**2.5 (2.8)	83.9 (5.3)	2.5 (2.8)	1.3 (2.6)	−3.8 (−4.1;11.7)	0.329	0.17
Disease duration ≥ 5	64.5 (5.5)	6.9 (3.7)	7.0 (5.4)	76.4 (4.2)	2.7 (2.8)	**−**1.3 (3.9)	8.2 (−21.7;5.2)	0.524	0.32
Age < 70	73.3 (4.4)	−0.6 (2.3)	−0.5 (3.5)	88.5 (3.5)	1.2 (1.8)	0.0 (2.7)	−0.5 (−8.5;9.6)	0.825	0.03
Age ≥ 70	67.2 (6.1)	4.1 (5.0)	4.4 (5.8)	68.3 (5.4)	4.0 (4.3)	**−**0.9 (4.9)	5.3 (−20.7;10.1)	0.295	0.21
No caregiver	80.9 (5.8)	4.6 (4.7)	7.2 (4.1)	90.1 (4.6)	1.6 (3.7)	**−**0.6 (3.3)	7.8 (−18.7;3.0)	0.126	0.29
Caregiver	65.3 (4.8)	−1.8 (2.7)	0.4 (4.8)	72.8 (4.2)	3.7 (2.3)	−0.3 (3.8)	0.8 (−13.1;11.5)	0.335	0.06

Data are analyzed by ‘Repeated measurement analysis’. Scores presented as means (SE). *BL* baseline score, Δ*BL-3 months* difference between 3 months and baseline. Δ*BL-6mnd* difference between 6 months and baseline. Effect M-C: difference ΔBL-6 months Medido – ΔBL-6 months Control.

a. *p*-value based on ‘time x measurement’ analysis of difference score between baseline and follow-up.

b. effect size: (effect M – C) / (SD of Medido group at baseline).

In exploratory sub analyses (Table 2), we observed a significant improvement in the Medido group compared to the control group (difference of 14.69 points (95% CI -28.5;-0.9) only in H&Y > 2.5. The effect size was 0.59. All other sub analyses revealed no significant differences.

#### Secondary outcomes

Data from the exploratory analyses are presented in Additional files 1, 2, 3, and 4.

##### PDQ-39

At baseline, significant differences between groups for the *total score* (*p* = 0.005), *mobility* (*p* = 0.018), *ADL* (*p* = 0.002) and *cognition* (*p* = 0.007) were in favor of the control group. After 6 months, both groups deteriorated in total PDQ score, but the Medido group deteriorated 1.0 point further (*p* = 0.01 for group x time interaction). Patients in the control group did numerically, but not significantly better, or deteriorated less in *emotional wellbeing*, *stigma*, *social support*, *communication,* and *physical wellbeing* domains compared to the Medido group, whereas the Medido group improved numerically, but not significantly in the *mobility*, *ADL* and *cognition* domains.

##### EQ5D-5L

The control group had a significant (*p* = 0.047) better initial EQ5D-5L score compared to the Medido group at baseline. After 6 months of follow-up both groups showed no change.

##### VAS-score

After 6 months, the Medido group improved by 0.4 points, while the control group remained stable (difference 0.4, 95% CI − 0.2;1.1, *p* = 0.057).

##### NMS-Quest

Baseline measurements showed significantly less non-motor symptoms (*p* = 0.016) in the control group than in the Medido group. After 6 months of follow-up, the Medido group deteriorated while the control group remained almost unchanged (difference 1.3, 95% CI − 0.5;3.0, *p* = 0.095 for group x time interaction).

##### PDQ-Carer

At baseline, the Medido group showed a significantly higher stress score (*p* = 0.01). We observed no differences in change over time between both groups in any of the domains in the PDQ-carer.

There were no modifications in LED after 6 months follow-up. We observed no harm or unintended effects related to the intervention.

## Discussion

This study is the first in assessing the effect of an electronic medication dispenser in PD patients, with the aim of improving functional disability by facilitating therapy adherence. A small, non-significant improvement in physical disability was seen after 6 months follow-up, as measured by the ALDS questionnaire. However, in a post-hoc sub analysis, a large improvement was seen in those with more advanced disease. What is unknown, however, is whether severity was the driving factor or whether greater improvement was possible due to there being more severe patients on more medications with more medication moments. No specific data on the number of medication moments were available. No significant differences were observed between both groups in most secondary outcomes.

Though various types of reminder systems and automated dispensing devices have been around for many years, there are very few studies of their use in PD patients. Approximately half of the studies show improvement in medication adherence, with over one-third reporting an improvement in clinical outcomes [22]. Only one study was performed in a group of 50 patients with advanced PD, showing that an SMS reminder system is a feasible method, i.e. 91% of the patients reported the system worked well for them. After a follow-up of 4 weeks, about half of the subjects experienced clear benefits, though therapy adherence or objective clinical outcomes were not measured [23]. We assumed that we would not be able to truly measure medication adherence, because patients were not checked for ingesting the medication and no blood or serum levels were assessed. Therefore, we used changes in clinical outcomes as a proxy for changes in therapy adherence. Our study revealed the added value of a medication dispenser box in more severe patients on functional disability. However, there was no benefit for the younger and less severe patients.

Research in advanced PD patients comes with specific challenges. Problems that patients experience with changing situations in their lives because of cognitive impairment, are well documented [11, 12]. Furthermore, reports suggest that over 20% of PD patients without dementia display evidence of cognitive impairment, with most commonly exhibiting executive dysfunction [24]. This may have contributed to the substantial drop out among the randomized participants (24/111 (22%) overall and 19/56 (34%) in the Medido group), before the study actually started. We accounted for a 10% dropout rate and, according to our protocol, individual subjects that withdrew from the study would not be replaced. Due to the larger than expected dropout rate, especially in the intervention group, our study was slightly underpowered in the end. Moreover, adaptation to the Medido may have been complex for those participants with H&Y stage > 2.5 in the Medido group. This may explain the modest improvement after 3 months and the bigger improvement after 6 months in the Medido group.

Not every aspect of PD was influenced by the Medido, but minor effects, more numerically than significant, in (domains of) questionnaires of motor-symptoms were seen, particularly in the older and more severe patient. NMS-Quest and the PDQ-carer questionnaires showed no differences in change over time between both groups. Non-motor symptoms have a mostly non-dopaminergic etiology and are therefore hard to treat. Also, non-motor symptoms frequently remain unrecognized by clinicians. Both might be explanations for the fact that the non-motor symptoms seem to be beyond the influence of the Medido [25]. In addition, in advanced PD, non-motor symptoms have a mainly negative effect on QoL. This could explain why we did not find an improvement of QoL as measured by the EQ5D-5L and PDQ-39 [26]. However, a non-significant improvement in the EQ-VAS score in the Medido group, may be explained by aspects of QoL that are important to people which are not reflected in the EQ5D-5L and PDQ-39 questionnaire domains [27]. Furthermore, it is understandable that carers do not experience noticeable effects of the Medido, which is not intended for them, but for their patients.

The Medido enables a vulnerable patient group to have longer independent use of their medication [14], in particular, when disability improves in patients with more advanced PD. In contrast, younger patients without complications do not seem to derive benefit from the Medido. This may be due to the smaller benefits that can be achieved because of experiencing fewer complaints. In addition, the Medido is not portable and may be a burden to the more active and outdoor oriented younger patient.

Limitations of the study include the many missing measurements at 3 months of follow-up as a result of logistic problems (illness of the senior researcher); this could have had an impact on outcome measurements. Also, therapy adherence was not directly measured, but rather by proxy using clinical outcomes. It is possible that the control group improved on therapy adherence because of inclusion in the study and knowledge of the questionnaires, known as the Hawthorne effect [28, 29]. Furthermore, the substantial and unforeseen levels of patient drop-out could explain the baseline differences, particularly in the secondary outcome measurements. Also, 35% of patients allocated to Medido did not start or continue the trial for unknown reasons.

## Conclusion

In conclusion, the electronic medication dispenser, Medido, does not have an impact on ADL in all PD patients, though it can offer an improvement in ADL in more advanced PD patients.

## Supplementary information

**Additional file 1.** Table with secondary outcomes of PDQ-39.

**Additional file 2.** Table with secondary outcomes of EQ5D-5L, VAS, NMS-Quest.

**Additional file 3.** Table with secondary outcomes of UPDRS-4.

**Additional file 4.** Table with secondary outcomes of PDQ-carer.

## Data Availability

The datasets used and or analyzed during the study are available from the corresponding author on reasonable request.

## Abbreviations

ADL: Activities of daily living
ALDS: AMC Linear Disability Scale
EQ5D-5L: EuroQol 5 dimensions 5 levels questionnaire
H&Y: Hoehn & Yahr classification
HRQoL: Health related quality of life
LED: Levodopa equivalent dose
NMS-Quest: Non-motor symptoms questionnaire
PD: Parkinson’s disease
PDQ-39: Parkinson’s disease quality of life questionnaire
PDQ-Carer: Parkinson’s disease quality of life questionnaire for carers
QoL: Quality of life
VAS: Visual Analogue Scale
