# Supplementary material for: A randomized controlled efficacy study of the Medido medication dispenser in Parkinson’s disease
Source: BMC Geriatr. 2019 Oct 16;19:273. doi: 10.1186/s12877-019-1292-y (PMC6796399; doi:10.1186/s12877-019-1292-y)
Supplement: Supplementary file 1 — Additional file 1. Table with secondary outcomes of PDQ-39. [file 12877_2019_1292_MOESM1_ESM.docx]

Additional File 1. Secondary outcomes: PDQ-39

| **PDQ-39**  [0-100] | **Medido** | | | | **Control** | | | | **Effect**  **M - C** | **P-value of**  **Difference scores ^+^** |
| --- | --- | --- | --- | --- | --- | --- | --- | --- | --- | --- |
|  | **BL (n=36)** | **3 months (n=24)** | **6 months (n=29)** | **ΔBL-6**  **months** | **BL (n=51)** | **3 months (n=36)** | **6 months (n=45)** | **ΔBL-6 months** | **Effect**  **(95%CI)** |  |
| **Total** | 40.2  (2.6) | 42.2  (2.8) | 44.4  (2.9) | **4.1**  (1.7) | 30.2  (2.2) | 34.8  (2.3) | 33.3  (2.4) | **3.1**  (2.2) | **1.0**  (-3.3; 5.3) | **0.010** |
| Mobility | 50.6  (4.1) | 48.7  (4.5) | 50.5  (4.5) | **0.1**  (2.8) | 37.4  (3.4) | 42.1  (3.8) | 39.9  (3.7) | **2.4**  (3.6) | **-2.6**  (-9.8;4.6) | **0.137** |
| ADL | 53.1  (4.0) | 51.7  (4.4) | 53.4  (4.2) | **0.3**  (2.4) | 36.4  (3.4) | 40.9  (3.6) | 41.0  (3.5) | **4.6**  (3.14) | **-4.8**  (-11.0; 1.5) | **0.542** |
| Emotional  wellbeing | 30.7  (3.0) | 37.0  (3.7) | 40.2  (3.5) | **9.5**  (2.6) | 23.5  (2.6) | 31.3  (3.1) | 29.5  (2.9) | **6.0**  (3.4) | **3.5**  (-3.2;10.2) | **0.075** |
| Stigma* | 12.5  (0.0;34.4) | 21.9  (0.0;39.1) | 25.0  (10.9;43.8) | **12.5** | 12.5  (0.0;20.3) | 12.5  (4.7;26.6) | 12.5  (0.0;25.0) | **0** | **12.5** | **0.125** |
|  |  |  |  |  |  |  |  |  |  |  |
| Social support* | 8.3  0.0;45.8 | 16.7  6.3;41.7 | 16.7  8.3;41.7 | **8.3** | 8.3  0.0;27.1 | 25.0  8.3;41.7 | 25.0  0.0;33.3 | **16.7** | **-8.3** | **0.261** |
|  |  |  |  |  |  |  |  |  |  |  |
| Cognition | 41.5  (3.2) | 43.5  (3.4) | 44.7  (3.2) | **3.2**  (2.1) | 29.8  (2.7) | 34.6  (2.9) | 34.4  (2.6) | **4.6**  (2.7) | **-1.4**  (-6.8; 4.1) | **0.431** |
| Communication | 36.6  (3.3) | 38.6  (3.3) | 38.6  (3.3) | **2.0**  (3.0) | 28.1  (2.8) | 28.0  (2.7) | 28.3  (2.7) | **0.2**  (3.9) | **1.7**  (-6.1; 9.6) | **0.854** |
| Physical  wellbeing | 43.1  (3.8) | 48.0  (3.8) | 49.9  (3.8) | **6.8**  (3.7) | 38.2  (3.2) | 41.5  (3.1) | 39.9  (3.1) | **1.9**  (4.7) | **5.1**  (-4.3; 14.6) | **0.449** |
| Table A1. Outcome PDQ-39 questionnaire data. Analysed by ‘Repeated measurement analysis’. Scores presented as means (SE). BL: baseline score, ΔBL-3mnd: difference between 3 months follow-up (FU) and baseline ΔBL-6mnd: difference between 6 months follow-up (FU) and baseline. Effect: difference ΔBL-6mnd intervention – ΔBL-6 months control. Lower score represents better outcome.  * Analysed by Mann-Whitney-U test. Scores presented as median (25-75 percentiles). Effect BL-6mnd: difference ΔBL-6mnd intervention – ΔBL-6mnd Control with p-value.  ^+^ p-value based on ‘group x time analysis of difference score between BL and FU, except *stigma* and *social support.* | | | | | | | | | |  |
