# Supplementary material for: A randomized controlled efficacy study of the Medido medication dispenser in Parkinson’s disease
Source: BMC Geriatr. 2019 Oct 16;19:273. doi: 10.1186/s12877-019-1292-y (PMC6796399; doi:10.1186/s12877-019-1292-y)
Supplement: Supplementary file 2 — Additional file 2. Table with secondary outcomes of EQ5D-5L, VAS, NMS-Quest. [file 12877_2019_1292_MOESM2_ESM.docx]

Additional File 2. Secondary outcomes: EQ5D, VAS, NMS-Quest

|  | **Medido** | | | | **Control** | | | | **Effect**  **M - C** | **P-value of**  **Difference scores ^+^** |
| --- | --- | --- | --- | --- | --- | --- | --- | --- | --- | --- |
|  | **BL (n=36)** | **3 months (n=24)** | **6 months (n=29)** | **ΔBL-6 months** | **BL (n=51)** | **3 months (n=36)** | **6 months (n=45)** | **ΔBL-6 months** | **Effect**  **(95%CI)** |  |
| **EQ5D**  [-0.33-1.00] | 0.62  (0.03) | 0.61  (0.03) | 0.59  (0.04) | **-0.02**  **(0.03)** | 0.70  (0.03) | 0.66  (0.03) | 0.67  (0.3) | **-0.04**  **(0.03)** | **0.01**  **(-0.06; 0.08)** | **0.475** |
|  |  |  |  |  |  |  |  |  |  |  |
| **VAS**  [0-10] | 6.3  (0.2) | 6.2  (0.2) | 6.7  (0.2) | **0.4**  **(0.3)** | 6.5  (0.2) | 6.6  (0.2) | 6.5  (0.2) | **-0.04**  **(0.32)** | **0.42**  **-0.22;1.05** | **0.057** |
| **NMS-Quest**  [0-30] | 12.4  (0.8) | 12.5  (0.8) | 13.6  (0.9) | **1.2**  **(0.7)** | 9.9  (0.6) | 10.1  (0.7) | 9.8  (0.7) | **-0.1**  **0.9** | **1.3**  **(-0.5; 3.0)** | **0.095** |
| Table A2. Outcome EQ5D, VAS and NMS questionnaire data. Analysed by ‘Repeated measurement analysis’. Scores presented as means (SE). BL: baseline score, ΔBL-6mnd: difference between 6 months and baseline. Effect BL-6mnd: difference ΔBL-6mnd intervention – ΔBL-6 months control. NMS-Quest: lower scores represents better outcome.  + p-value based on ‘time x measurement’ analysis of difference score between baseline and follow-up. | | | | | | | | | | |
