# Supplementary material for: A randomized controlled efficacy study of the Medido medication dispenser in Parkinson’s disease
Source: BMC Geriatr. 2019 Oct 16;19:273. doi: 10.1186/s12877-019-1292-y (PMC6796399; doi:10.1186/s12877-019-1292-y)
Supplement: Supplementary file 3 — Additional file 3. Table with secondary outcomes of UPDRS-4. [file 12877_2019_1292_MOESM3_ESM.docx]

Additional File 3. Secondary outcomes: UPDRS-4

| **UPDRS-4**  [0-4] | **Medido** | | | | **Control** | | | | **Effect**  **M - C** | **P-value of**  **Difference scores ^+^** |
| --- | --- | --- | --- | --- | --- | --- | --- | --- | --- | --- |
|  | **BL (n=36)** | **3 months (n=24)** | **6 months (n=29)** | **ΔBL-6 months** | **BL (n=51)** | **3 months (n=36)** | **6 months (n=45)** | **ΔBL-6 months** | **Effect**  **(95%CI)** |  |
| Time dyskinesias | 0.78  (0.15) | 0.55  (0.15) | 0.56  (0.16) | **-0.21**  (0.19) | 0.68  (0.13) | 0.69  (0.3) | 0.67  (0.13) | **0.00**  (0.24) | **-0.20**  (-0.7;0.3) | **0.771** |
| Functional impact of dyskinesias | 0.53  (0.15) | 0.77  (0.18) | 0.44  (0.17) | **-0.09**  (0.20) | 0.62  (0.13) | 0.62  (0.16) | 0.52  (0.14) | **-0.09**  (0.26) | **0.00**  (-0.5;0.5) | **0.391** |
| Time in  off-phase | 1.47  (0.13) | 1.24  (0.13) | 0.86  (0.11) | **-0.61**  (0.16) | 1.46  (0.11) | 1.24  (0.11) | 1.00  (0.09) | **-0.54**  (0.22) | **-0.15**  (-0.6;0.3) | **0.769** |
| Functional consequences  of fluctuations | 2.42  (0.20) | 2.33  (0.25) | 1.71  (0.25) | **-0.71**  (0.25) | 2.08  (0.17) | 2.98  (0.21) | 1.77  (0.20) | **-0.29**  (0.33) | **-0.41**  (-1.1;0.2) | **0.222** |
| Complexity motor complications | 3.11  (0.20) | 2.73  (0.23) | 1.80  (0.25) | **-1.31**  (0.31) | 2.60  (0.17) | 2.11  (0.20) | 2.06  (0.20) | **-0.46**  (0.39) | **-0.78**  (-1.6;0.0) | **0.105** |
| Painful dystonias in off phase | 1.03  (0.17) | 0.96  (0.18) | 0.54  (0.21) | **-0.48**  (0.22) | 0.98  (0.15) | 0.70  (0.15) | 0.66  (0.16) | **-0.28**  (0.28) | **-0.17**  (-0.7;0.4) | **0.177** |
| Table A3. Outcome PDQ-39 questionnaire data. Analysed by ‘Repeated measurement analysis’. Scores presented as means (SE). BL: baseline score, ΔBL-6mnd: difference between 6 months and baseline. Effect BL-6mnd: difference ΔBL-6mnd intervention – ΔBL-6 months control.  ^+^ p-value based on ‘time x measurement’ analysis of difference score between baseline and follow-up. | | | | | | | | | |  |
