# Supplementary material for: A randomized controlled efficacy study of the Medido medication dispenser in Parkinson’s disease
Source: BMC Geriatr. 2019 Oct 16;19:273. doi: 10.1186/s12877-019-1292-y (PMC6796399; doi:10.1186/s12877-019-1292-y)
Supplement: Supplementary file 4 — Additional file 4. Table with secondary outcomes of PDQ-carer. [file 12877_2019_1292_MOESM4_ESM.docx]

Additional File 4. Secondary outcomes: PDQ-carer

| **PDQ-carer**  [0-100] | **Medido** | | | | **Control** | | | | **Effect**  **M - C** | **P-value of**  **Difference scores ^+^** |
| --- | --- | --- | --- | --- | --- | --- | --- | --- | --- | --- |
|  | **BL (n=25)** | **3 months (n=14)** | **6 months (n=16)** | **ΔBL-6 months** | **BL (n=33)** | **3 months (n=19)** | **6 months (n=23)** | **ΔBL-6 months** | **Effect**  **(95%CI)** |  |
| **Total** | 38.2  (3.5) | 36.8  (3.8) | 36.6  (3.5) | **-1.6**  (2.5) | 28.8  (3.0) | 28.1  (3.2) | 28.2  (3.0) | **-0.6**  (3.3) | **-1.1**  (-7.7; 5.5) | **0.867** |
| Personal, social activities | 37.3  (3.4) | 39.1  (3.9) | 41.6  (3.5) | **4.4**  (2.6) | 30.0  (2.9) | 29.4  (3.3) | 28.9  (2.9) | **-1.1**  (3.4) | **5.5**  (-1.4; 12.3) | **0.290** |
| Anxiety, depression | 29.7  (3.8) | 25.8  (3.3) | 29.2  (3.7) | **-0.5**  (2.9) | 23.1  (3.3) | 23.5  (2.8) | 22.3  (3.1) | **-0.8**  (3.8) | **0.3**  (-7.4; 7.9) | **0.529** |
| Self-care | 42.2  (4.3) | 37.1  (4.8) | 31.9  (4.3) | **-10.3**  (3.5) | 31.3  (3.7) | 28.4  (4.1) | 28.9  (3.6) | **-2.4**  (4.6) | **-7.9**  (-17.2; 1.4) | **0.188** |
| Stress | 45.5  (4.2) | 43.0  (5.5) | 37.9  (4.6) | **-7.6**  (3.7) | 30.3  (3.6) | 30.1  (4.6) | 32.1  (3.9) | **1.8**  (4.9) | **-9.4**  (-19.2; 0.4) | **0.163** |
| Table A4. Outcome PDQ-Carer questionnaire data. Analysed by ‘Repeated measurement analysis’. Scores presented as means (SE). BL: baseline score, ΔBL-6mnd: difference between 6 months and baseline. Effect BL-6mnd: difference ΔBL-6mnd intervention – ΔBL-6 months control.  ^+^ p-value based on ‘time x measurement’ analysis of difference score between baseline and follow-up | | | | | | | | | |  |
